# Sample size re-estimation in crossover trials: application to the AIM HY-INFORM study

**DOI:** 10.1186/s13063-019-3724-6

**Published:** 2019-12-02

**Authors:** Julie Wych, Michael J. Grayling, Adrian P. Mander

**Affiliations:** 1 Medical Research Council Biostatistics Unit, University of Cambridge, School of Clinical Medicine, Forvie Site, Robinson Way, Cambridge, CB2 0SR UK; 2 Institute of Health & Society, Newcastle University, Baddiley-Clark Building, Richardson Road, Newcastle upon Tyne, NE2 4AX UK; 3 Centre for Trials Research, College of Biomedical & Life Sciences, Cardiff University, 7th Floor, Neuadd Meirionnydd, Heath Park, Cardiff, CF14 4YS UK

**Keywords:** Crossover trial, Sample size calculation, Simulation study, AIM HY-INFORM study, Hypertension

## Abstract

**Background:**

Crossover designs are commonly utilised in randomised controlled trials investigating treatments for long-term chronic illnesses. One problem with this design is its inherent repeated measures necessitate the availability of an estimate of the within-person standard deviation (SD) to perform a sample size calculation, which may be rarely available at the design stage of a trial. Interim sample size re-estimation designs can be used to help alleviate this issue by adapting the sample size mid-way through the trial, using accrued information in a statistically robust way.

**Methods:**

The AIM HY-INFORM study is part of the Informative Markers in Hypertension (AIM HY) Programme and comprises two crossover trials, each with a planned recruitment of 600 participants. The objective of the study is to test whether blood pressure response to first line antihypertensive treatment depends on ethnicity. An interim analysis is planned to reassess the assumptions of the planned sample size for the study. The aims of this paper are: (1) to provide a formula for sample size re-estimation in both crossover trials; and (2) to present a simulation study of the planned interim analysis to investigate alternative within-person SDs to that assumed.

**Results:**

The AIM HY-INFORM protocol sample size calculation fixes the within-person SD to be 8 mmHg, giving > 90% power for a primary treatment effect of 4 mmHg. Using the method developed here and simulating the interim sample size reassessment, if we were to see a larger within-person SD of 9 mmHg at interim, 640 participants for 90% power 90% of the time in the three-period three-treatment design would be required. Similarly, in the four-period four-treatment crossover design, 602 participants would be required.

**Conclusions:**

The formulas presented here provide a method for re-estimating the sample size in crossover trials. In the context of the AIM HY-INFORM study, simulating the interim analysis allows us to explore the results of a possible increase in the within-person SD from that assumed. Simulations show that without increasing the planned sample size of 600 participants, we can reasonably still expect to achieve 80% power with a small increase in the within-person SD from that assumed.

**Trial registration:**

ClinicalTrials.gov, NCT02847338. Registered on 28 July 2016.

## Background

Randomised crossover trials are a well-established design for long-term chronic illnesses such as hypertension [1]. The UK hypertension NICE guidance (CG127) stratifies hypertension treatment by age and self-defined ethnicity (SDE), with guidelines adopting a ‘black versus white’ approach [2]. The problems with this stratification include a lack of data from UK populations supporting the current SDE stratification and no reference to South Asians – the largest ethnic minority group in the UK [2]. Consequently, the primary objective of the AIM HY-INFORM study is to determine if the response to existing first-line antihypertensive drugs differs by ethnic group, white British, black African/African Caribbean or South Asian, for participants who are newly diagnosed or established hypertensives.

The AIM HY-INFORM study comprises two open-label, randomised crossover trials: one three-period three-treatment (monotherapy) trial for participants newly diagnosed with hypertension and one four-period four-treatment (dual therapy) trial for participants with existing hypertension. The primary outcome is systolic blood pressure (SBP) mmHg; linear mixed models are the preferred method of analysis for a crossover design with a continuous outcome variable [1]. Repeated measurements of SBP taken from the same participant are correlated and this correlation needs to be accounted for in sample size calculations. That is, sample size estimation for any repeated measures design requires an estimate of the within-person standard deviation (SD). Taking estimates of the within-person SD from other studies can be unreliable due to differences in the study population and participant attributes, instruments and measurement techniques, or other background conditions, which can result in trials that are either under or over-powered [3, 4]. With the absence of reliable prior estimates of the within-person SD available for the AIM-HY INFORM study, in particular for South Asian ethnicities, the calculation of the required sample size to ensure the desired power to detect a single treatment-by-ethnic interaction is challenging.

To address this issue in many repeated measures contexts, sample size re-estimation designs have been considered [3, 5–7]. However, there is little, directly applicable work on sample size re-estimation at interim for crossover designs. Zucker and Denne describe a strategy to deal with the unknown within-person SD in a two-stage, repeated measures design that examines the accrued data at an interim point, obtaining an estimate of the within-person SD. They then use this estimate to update the covariance parameter in the linear mixed model and modify the sample size required to ensure the trial has sufficient power [3]. Moreover, a collection of papers have addressed sample size re-estimation in bioequivalence trials using a two-period two-treatment crossover design [8–11]. While more recently, methodology for blinded and unblinded sample size re-estimation in multi-treatment crossover trials balanced for period was described [12]. None of these papers, however, directly allows for re-estimation in the context of the AIM HY-INFORM study, for reasons that will be described shortly.

Here, we present the framework for sample size re-estimation in both our 3 × 3 (monotherapy) and 4 × 4 (dual therapy) settings. Precisely, the study design and models used are described, along with the methods developed for re-estimation of the required sample size. The planned interim analysis in the AIM HY-INFORM study states in the protocol that after 50 individuals have completed their treatment sequence, the sample size calculation for both the mono and dual-therapy treatment rotations will be recalculated using a mid-trial estimate of the within-person SD. Therefore, following our initial descriptions, results from a simulation study carried out ahead of trial recruitment, with the aim of simulating the interim analysis to explore the effect of a larger within-person SD from that assumed in the protocol are presented.

## Methods

### Study design

AIM HY-INFORM is a multicentre, prospective study comprising two randomised, open-label crossover trials (three-period three-treatment monotherapy and four-period four-treatment dual therapy) in a multi-ethnic cohort of hypertensive participants, where each study requires separate randomisation to treatment sequences [2].

Participants on both trials self-identify into one of the following three ethnic groups (SDE):

- White (white British, white Irish or any other white background);
- Black or black British (black Caribbean, black African or any other black background);
- Asian or Asian British (Asian Indian, Asian Pakistani, Asian Bangladeshi or any other South Asian background).

**Fig. 1:**
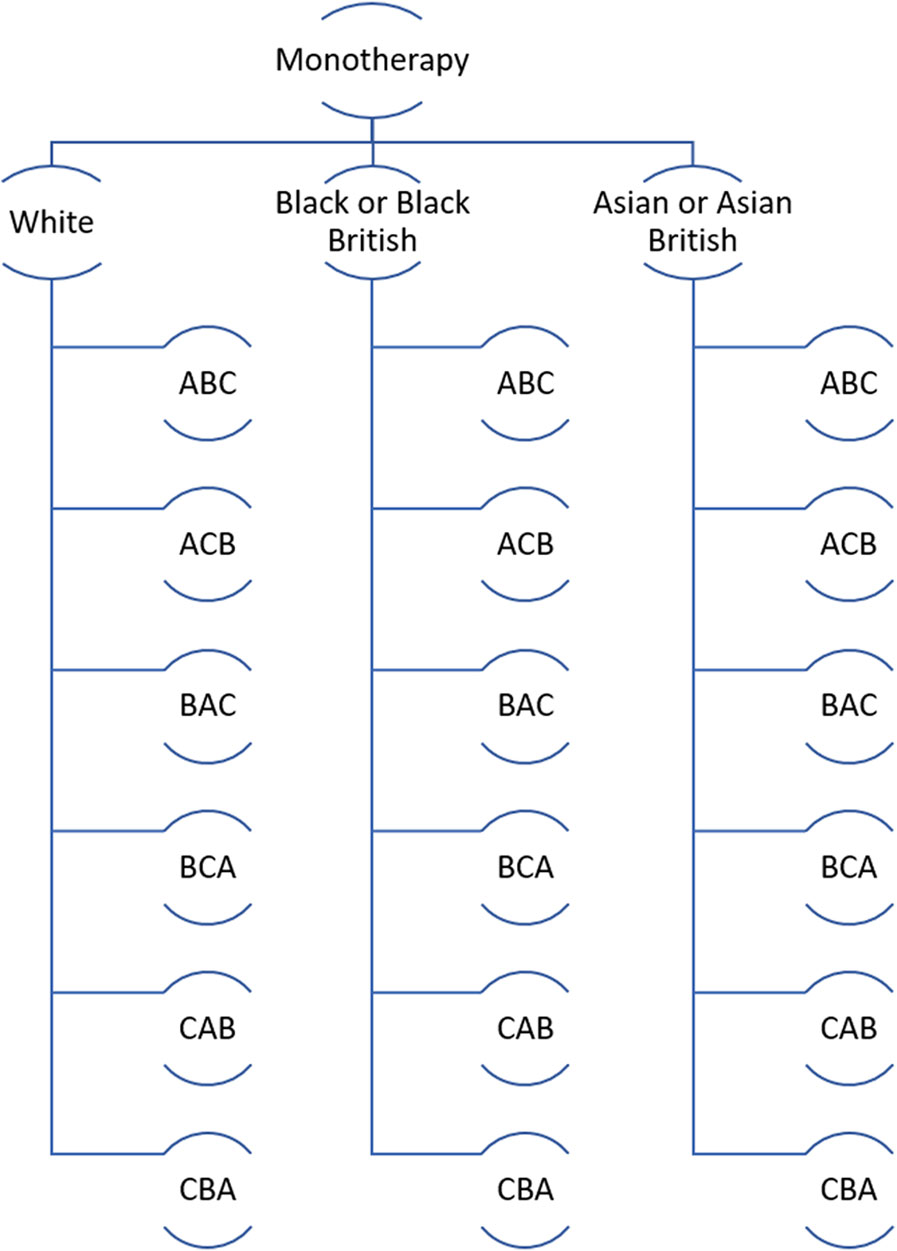
Participants receive each treatment A, B and C; they are randomised to a treatment sequence according to two three-period three-treatment Latin square designs: ABC, ACB, BAC, BCA, CAB and CBA, balanced by ethnic group

The monotherapy study is a 24-week three-period three-treatment crossover trial of newly diagnosed hypertensives with Ambulatory Blood Pressure Monitoring (ABPM) ≥ 135/85 mmHg. After initial screening and baseline measurement collection, participants are randomised with equal allocation to one of six sequences of treatments from two three-period three-treatment Latin square designs: ABC; ACB; BAC; BCA; CAB; and CBA [13]. Here, treatment A is 1–2 weeks of Amlodipine 5 mg followed by 6 –7 weeks of Amlodipine 10 mg, treatment B is 1–2 weeks of Lisinopril 10 mg followed by 6–7 weeks of Lisinopril 20 mg and treatment C is approximately eight weeks of 25 mg Chlortalidone (Fig. 1).

The dual-therapy study is a 32-week four-period four-treatment crossover trial of established hypertensives with ABPM > 135/85 and < 200/110. After initial screening and baseline measurement collection, participants are randomised with equal allocation to one of four sequences of treatments from a four-treatment four-period Williams square design: ABDC; BCAD; CDBA; and DACB. There are 24 possible Latin squares for a four-treatment crossover design; the design used here is one of six special cases of the Latin square design which are balanced for first-order carry-over and are known as Williams Squares [13, 14].

**Fig. 2:**
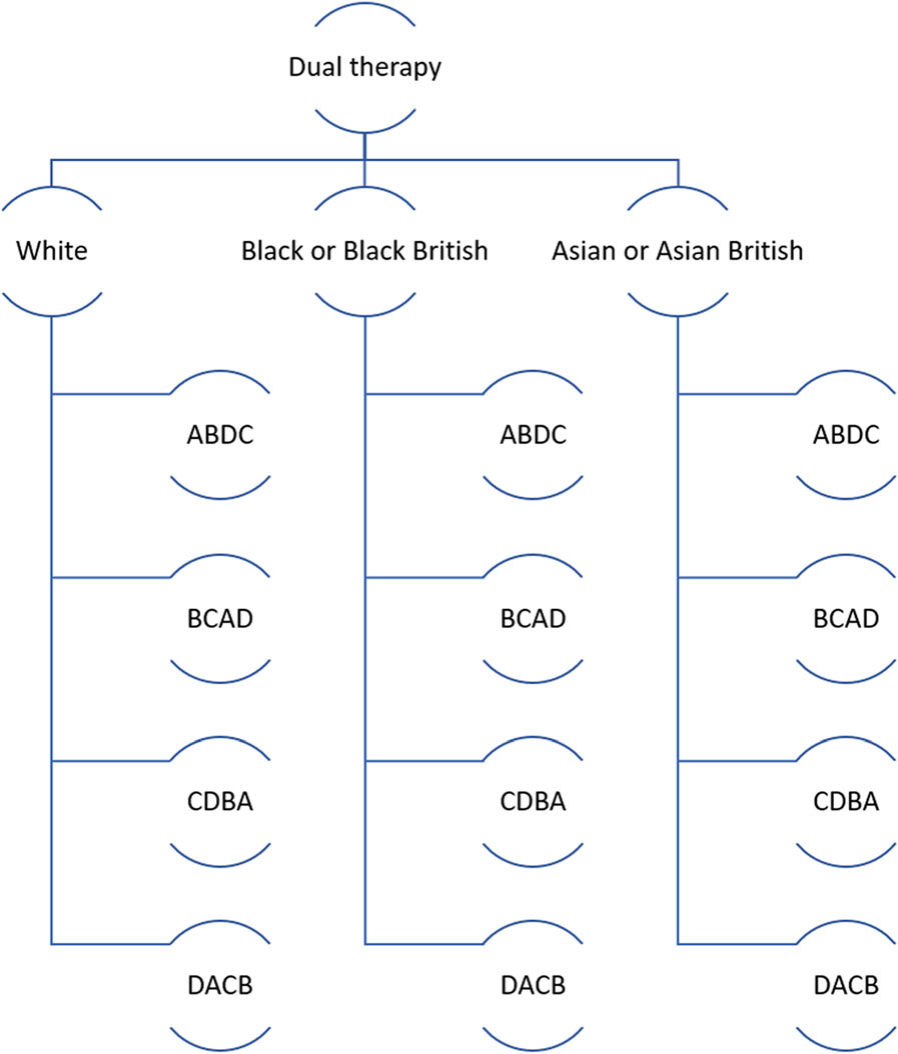
Participants receive each treatment A, B, C and D; they are randomised to a treatment sequence according to a four-period four-treatment Williams square design: ABDC, BCAD, CDBA and DACB, balanced by ethnic group

For participants on dual therapy, treatment A is eight weeks of Amlodipine 5 mg and Lisinopril 20 mg, treatment B is eight weeks of Amlodipine 5 mg and Chlortalidone 25 mg, treatment C is eight weeks of Lisinopril 20 mg and Chlortalidone 25 mg and treatment D is eight weeks of Amiloride 10 mg and Chlortalidone 25 mg (Fig. 2).

The two randomised crossover trials (monotherapy and dual therapy) are open-label and require separate randomisation to treatment sequence. To control for balance permuted blocks within strata are implemented with an allocation ratio: 1:1:1:1:1:1 for the monotherapy trial and 1:1:1:1 for the dual-therapy trial.

The monotherapy and dual-therapy trials are distinct and analysed using separate linear mixed models. The primary outcome of both trials is seated automated office SBP mmHg as measured eight weeks (± 4 days) after receiving each treatment. The primary objective of the trials is to test for a significant treatment-by-ethnic group interaction.

With the absence of an available estimate of the within-participant SD for the trial participants, the protocol estimates a sample size of 600 participants assuming a fixed within-person SD of 8 mmHg, based on previous clinical trial data in people representative of the general population with either high normal blood pressure or mild hypertension [2]. More precisely, for a within-person SD of 8 mmHg and a single treatment-by-ethnic interaction of 3 mmHg, with all other interactions being 0 mmHg, the protocol outlines that a sample size of 600 participants produces 81.3% power to detect treatment-by-ethnic interactions using a global test of interaction at a 5% significance level. For the same fixed within-person SD of 8 mmHg and a larger single treatment-by-ethnic interaction of 4 mmHg, a sample size of 600 participants would give 98% power to detect a single treatment-by-ethnic interaction. A 10% over-recruitment allows for loss to follow-up, resulting in 220 planned enrolments from each ethnic group, for each trial [3].

In order to check the assumptions that the within-person SD is 8 mmHg, there is a planned interim analysis after approximately 50 participants have completed their treatment sequence. The aim of the interim analysis is twofold: (1) to obtain an estimate of the within participant SD from trial participants; and (2) to use this estimate to calculate the sample size required for either 80 or 90% power to detect a treatment-by-ethnic interaction using a global test of interaction at a 5% significance level. The aim here is to describe the method for the sample size re-estimation and present results from a simulation study carried out ahead of the interim analysis.

Sample size calculations for the protocol along with simulations, sample size and power calculations for the interim analysis were carried out using Stata Statistical Software: Release 14 (StataCorp LP, College Station, TX, USA).

### Models and sample size calculations

#### Sample size re-estimation in the three-period three-treatment monotherapy crossover

The linear mixed effects model for the three-period three-treatment crossover study has fixed effects for treatment, period, ethnic group and treatment-by-ethnic group interactions. Additionally, subject is included as a random effect. This is our unrestricted model. We compare this unrestricted model with a nested restricted model that does not contain the treatment-by-ethnic group interaction terms.

Restricted model, assuming *n* participants are recruited in total:

$$ {y}_{ijkl}=\mu +{\tau}_{\mathrm{d}\left(j,k\right)}+{\pi}_j+{e}_l+{u}_{ikl}+{\epsilon}_{ijkl}. $$

Unrestricted model:

$$ {y}_{ijkl}=\mu +{\tau}_{\mathrm{d}\left(j,k\right)}+{\pi}_j+{e}_l+{\theta}_{\mathrm{d}\left(j,k\right)l}+{u}_{ikl}+{\epsilon}_{ijkl}. $$

Here

- *i* = 1, …, *n*/18 indicates a particular individual, *j* = 1, 2, 3 indicates the time period, *k* = 1, …, 6 indicates the sequence a particular individual was allocated and *l* = 1, 2, 3 indicates which of the three ethnicities a particular individual self-defined as. That is, *i*, *k* and *l* together completely prescribe a particular individual in the trial (there are *n* unique combinations of these three indices);
- *μ* is an intercept term (the mean of the values *y*_*i*1*k*1_);
- *τ*_d(*j*, *k*)_ is the direct effect of the treatment administered to a participant on sequence *k* in period *j*. That is, d(*j*, *k*) ∈ {*A*, *B*, *C*}. For identifiability purposes, we set *τ*_*A*_ = 0;
- *π*_*j*_ is a fixed effect for period, with *π*_1_ = 0 for identifiability;
- *e*_*l*_ is a fixed effect for ethnic group, with *e*_1_ = 0 for identifiability;
- *θ*_d(*j*, *k*)*l*_ is a fixed interaction effect for treatment d(*j*, *k*) by ethnic group *l*. For identifiability, we set *θ*_*A*1_ = *θ*_*A*2_ = *θ*_*A*3_ = *θ*_*B*1_ = *θ*_*C*1_ = 0.
- ${u}_{ikl}\sim {N}_1\left(0,{\sigma}_b^2\right)$ is a random participant effect;
- ${\epsilon}_{ijkl}\sim {N}_1\left(0,{\sigma}_e^2\right)$ is the residual error.

We perform a global Wald test to see if the unrestricted model gives a significantly better fit than the restricted, in order to test the primary hypothesis of whether there is an interaction between treatment and ethnic group. To perform sample size re-estimation, we require the sampling distribution of a suitable test statistic under the null and alternative hypotheses.

Precisely, setting ***θ*** = (*θ*_*B*2_, *θ*_*B*3_, *θ*_*C*2_, *θ*_*C*3_)^⊤^, we test the following null hypothesis of no treatment-by-ethnic interactions

$$ {H}_0:\boldsymbol{\theta} =\mathbf{0}, $$

against the following alternative

$$ {H}_1:\boldsymbol{\theta} \ne \mathbf{0}. $$

Denoting by $\hat{\boldsymbol{\theta}}={\left({\hat{\theta}}_{B2},{\hat{\theta}}_{B3},{\hat{\theta}}_{C2},{\hat{\theta}}_{C3}\right)}^{\top }$ our estimates of the interaction terms computed using conventional maximum likelihood estimation, we can test *H*_0_ using the following test statistic

$$ t={\hat{\boldsymbol{\theta}}}^{\top }\ \hat{\mathbf{Cov}}{\left(\hat{\boldsymbol{\theta}}\right)}^{-1}\hat{\boldsymbol{\theta}}. $$

Moreover, we power our trial for an alternative in which there is a single non-zero treatment-by-ethnic interaction (*θ*_*B*2_ = *δ*, i.e. ***θ*** = ***δ*** = (*δ*, 0, 0, 0)^⊤^).

The complexity of performing a hypothesis test in this setting, either in a fixed design or following sample size re-estimation, arises from the fact that the sampling distribution of *T*, the random unknown value of *t*, is in general complex to compute. Explicitly, while the numerator degrees of freedom in a suitable *F*-test would be 4, the denominator degrees of freedom is difficult to assign. Kenward and Roger [15] provided a comprehensive solution to this problem for fixed sample designs by computing the exact denominator degrees of freedom, but unfortunately their approach does not lend itself naturally to sample size re-estimation, as the degrees of freedom specification procedure is data-dependent.

Accordingly, Grayling et al. [12] specified the denominator degrees of freedom as that of a corresponding multi-level single-stage ANOVA design. For our unrestricted model this would designate the denominator degrees of freedom, *ν*, for sample size *n*, as

$$ \nu =3n-n-10=2n-10. $$

Here, the 3*n* term arises as the total number of measurements accrued, while 1 degree of freedom is subtracted for each participant, and for each fixed effect in the model. Thus, at the interim we suppose that we would reject *H*_0_ at the end of the trial when *t* > *F*^−1^(1 − *α*, 0, 4, 2*n* − 10), where *F*^−1^(*q*, 0, *m*, *n*) is the 100*q* th quantile of an *F*(0, *a*, *b*)-distribution (a central *F*-distribution on *a* and *b* degrees of freedom). Combining this with a suitable non-central *F*-distribution under the chosen alternative at which the trial is to be powered, interim re-estimation can then be performed.

Such an approach, however, was found by Grayling et al. [12] to, in many circumstances, lead to a notable inflation of the type-I error rate, and frequently provide power slightly below the desired level under the specified alternative. Therefore, they discussed the utility of a potential *α*-adjustment procedure, of using the above methodology for interim re-estimation but performing the final hypothesis test using the method of Kenward and Roger [15], and they explored the advantages of a sample size inflation factor. A problem with these adjustments as individual amendments to the basic re-estimation procedure described above, however, is that predicting their impact on both the type-I error rate and the power can be difficult.

Consequently, here, desiring to accurately control the type-I error rate while maintaining a high level of power, we make a heuristic conservative adjustment to the above degrees of freedom based on Hotelling’s *T*^2^ distribution [16].

Precisely, for ***x***_1_, …, ***x***_*n*_~*N*_*p*_(***ϕ***, **Ω**), the test statistic ${t}^2={\left(\overline{\boldsymbol{x}}-\boldsymbol{\phi} \right)}^{\top}\hat{Cov}{\left(\overline{\boldsymbol{x}}\right)}^{-1}\left(\overline{\boldsymbol{x}}-\boldsymbol{\phi} \right)\sim {T}^2\left(p,n-1\right)$, Hotelling’s *T*^2^ distribution on *p* and *n* − 1 degrees of freedom. We can work with this distribution using standard statistical software via the following relationship

$$ {T}^2\left(p,n-1\right)=\frac{p\left(n-1\right)}{n-p}F\left(p,n-p\right). $$

In our case, we therefore suppose *H*_0_ will be rejected when

$$ \left\{\frac{2n-13}{4\left(2n-10\right)}\right\}t>{F}^{-1}\left(1-\alpha, 4,2n-13\right). $$

Moreover, to attain power 1 − *β* we can search for the minimal integer *n* such that

$$ \mathbb{P}\left[\left\{\frac{2n-13}{4\left(2n-10\right)}\right\}T>{F}^{-1}\left(1-\alpha, 4,2n-13\right)\right]\ge 1-\beta, $$

for

$$ T\sim F\left\{{\boldsymbol{\delta}}^{\top}\hat{\mathbf{Cov}}{\left(\hat{\boldsymbol{\theta}}\right)}^{-1}\boldsymbol{\delta}, 4,2n-13\right\}. $$

Such a search is easy to perform using interval bisection over the discrete *n*. The final problem therefore is to specify $\hat{\mathbf{Cov}}{\left(\hat{\boldsymbol{\theta}}\right)}^{-1}$. In the Appendix, we justify the use of

$$ \hat{\mathbf{Cov}}\left(\hat{\boldsymbol{\theta}}\right)=\frac{12{\hat{\sigma}}_e^2}{n}\left(\begin{array}{cccc}1& 0.5& 0.5& 0.25\\ {}0.5& 1& 0.25& 0.5\\ {}0.5& 0.25& 1& 0.5\\ {}0.25& 0.5& 0.5& 1\end{array}\right), $$

where ${\hat{\sigma}}_e^2$ is the interim estimated within person SD, computed using REML (for the reasons outlined below).

With this, our methodology for re-estimating the required sample size in the 3 × 3 trial is complete. However, we must still specify how the final analysis will be performed.

Here, we use REML estimation as it takes into account the uncertainty in the fixed parameters in the model into account when estimating the random parameters, in theory leading to better estimates of the variance components with reduced bias when the number of groups is small [17]. Additionally, we use the Kenward–Roger approximation [15] to compute the denominator degrees of freedom in the final *F*-test. These choices are again made in order to limit the possibility of observing inflation to the type-I error rate. As AIM HY-INFORM is a confirmatory trial of treatment differences between different ethnic groups, inflation of the type-I errors should be avoided.

#### Sample size re-estimation in the four-period four-treatment dual-therapy crossover

The dual-therapy trial is a four-period four-treatment crossover. It will compare the same restricted and unrestricted models as for the three-period three-treatment monotherapy trial. However, we now have

- *i* = 1, …, *n*/12, *j* = 1, …, 4, *k* = 1, …, 4, and *l* = 1, 2, 3;
- d(*j*, *k*) ∈ {*A*, *B*, *C*, *D*};
- For identifiability, we set *θ*_*A*1_ = *θ*_*A*2_ = *θ*_*A*3_ = *θ*_*B*1_ = *θ*_*C*1_ = *θ*_*D*1_ = 0.

Here, setting ***θ*** = (*θ*_*B*2_, *θ*_*B*3_, *θ*_*C*2_, *θ*_*C*3_, *θ*_*D*2_, *θ*_*D*3_)^⊤^, we again test the following hypotheses

$$ {H}_0:\boldsymbol{\theta} =\mathbf{0},{H}_1:\boldsymbol{\theta} \ne \mathbf{0}. $$

For $\hat{\boldsymbol{\theta}}={\left({\hat{\theta}}_{B2},{\hat{\theta}}_{B3},{\hat{\theta}}_{C2},{\hat{\theta}}_{C3},{\hat{\theta}}_{D2},{\hat{\theta}}_{D3}\right)}^{\top }$ our test is once more based upon

$$ T={\hat{\boldsymbol{\theta}}}^{\top }\ \hat{\mathbf{Cov}}{\left(\hat{\boldsymbol{\theta}}\right)}^{-1}\hat{\boldsymbol{\theta}}. $$

Applying the Hotelling’s *T*^2^ based adjustment described above, in this case at interim we suppose *H*_0_ will be rejected when

$$ \left\{\frac{3n-19}{6\left(3n-14\right)}\right\}t>{F}^{-1}\left(1-\alpha, 6,3n-14\right), $$

and choose the required sample size by determining the minimal *n* such that

$$ \mathbb{P}\left[\left\{\frac{3n-19}{6\left(3n-14\right)}\right\}T>{F}^{-1}\left(1-\alpha, 6,3n-14\right)\right]\ge 1-\beta, $$

for

$$ T\sim F\left\{{\boldsymbol{\delta}}^{\top}\hat{\mathbf{Cov}}{\left(\hat{\boldsymbol{\theta}}\right)}^{-1}\boldsymbol{\delta}, 6,3n-14\right\}, $$

with ***δ*** = (*δ*, 0, 0, 0, 0, 0)^⊤^, and where in the Appendix we now justify the use of

$$ \hat{\mathbf{Cov}}\left(\hat{\boldsymbol{\theta}}\right)=\frac{12{\hat{\sigma}}_e^2}{n}\left(\begin{array}{cccccc}1& 0.5& 0.5& 0.5& 0.25& 0.25\\ {}0.5& 1& 0.5& 0.25& 0.5& 0.25\\ {}0.5& 0.5& 1& 0.25& 0.25& 0.5\\ {}0.5& 0.25& 0.25& 1& 0.5& 0.5\\ {}0.25& 0.5& 0.25& 0.5& 1& 0.5\\ {}0.25& 0.25& 0.5& 0.5& 0.5& 1\end{array}\right). $$

Finally, as for the 3 × 3 trial, the final analysis is performed using REML estimation and the Kenward–Roger correction.

At the interim, 50 participants have completed either three treatment periods if they are on the monotherapy trial, or four treatment periods if they are on the dual-therapy trial. Inherently, we have more data from the four-treatment crossover and may anticipate better performance in this setting. It is important to note that at this stage we are not concerned with estimating treatment effects, we simply want the estimate, ${\hat{\sigma}}_e^2$, of the within-person variance required for the sample size calculations outlined above.

### Simulation design

To consider the variation in the estimate of the within-person SD, a simulation study was carried out to investigate the effect on the requisite final sample size for different treatment effects and within-person SD scenarios, based on the sample size re-estimation calculations described above. In the protocol, the sample size calculation fixes the SD to be 8 mmHg giving power equal to 98% for a single 4 mmHg interaction effect with 600 participants, and 81.3% power with 600 participants and a single 3 mmHg interaction effect.

Two separate simulation studies have been carried out assuming participants are on either the monotherapy or dual-therapy treatment regimes, in all cases assuming that each participant has received three (monotherapy) or four (dual-therapy) treatments and that there are no missing values. Participants are randomly generated with approximately equal numbers of participants on each of the six treatment sequences—ABC, ACB, BAC, BCA, CAB and CBA—for monotherapy and four treatment sequences—ABDC, BCAD, CDBA and DACB—for dual therapy. We consider scenarios in which 80% and 90% power to detect treatment-by-ethnic interactions are desired, using our outlined global test of interaction at a 5% significance level are assumed.

What is important to realise is that in sample size calculations the within-person SD is typically fixed. In our simulation study, we set the desired power, the strength of a single treatment-by-ethnic interaction and a within-person SD, and solve for sample size across numerous replicates. Accordingly, of principle interest is the distribution of the interim estimated values of the within-participant SD and the distribution of the final required sample sizes.

The factors varied and held constant in these simulations are outlined in Table 1, resulting in 2 × 2 = 4 scenarios to assess for each of the two trials, with 1000 simulations carried out for each scenario-trial combination. We assume that there is no carryover effect and no treatment-by-period interaction. The between-person SD is held constant at 10 mmHg, we do not need to consider varying this as the procedures should be invariant to *σ*_*b*_ with a sample size of 50.

**Table 1:** Fixed and constant parameter values for the interim analysis simulations

Description	Constant factors	Factors varied
Interim sample size	*n* = 50	
Minimum required sample size	*n* = 600	
Number of simulations	1000	
Between-person SD	*σ*_*b*_ = 10 mmHg	
Within person SD		*σ*_*e*_ = 9, 10 mmHg
Overall mean SBP (mmHg)	*μ* = 140 mmHg	
Single treatment-by-ethnic interaction (mmHg)		*δ* = 3, 4 mmHg
Period effect (mmHg)	*π*_*j*_ = 0 mmHg, ∀*j*	
Power to detect a single ethnic by treatment interaction at 5% significance level (%)		1 − *β* = 0.1,0.2

Our methodology for re-estimating the sample size requires a minimum acceptable sample size. In the protocol, a sample size of 600 is estimated for each monotherapy and dual-therapy trial, based on a within-person SD of 8 mmHg. As there is no plan to reduce the sample size at interim, we therefore set a minimum sample size of 600 for all simulations and scenarios investigated. Matlab and Stata code for sample size calculations can be found in Additional files 1, 2, 3 and 4.

## Results

### Simulation of the interim analysis and re-estimation of the sample size: monotherapy

**Fig. 3:**
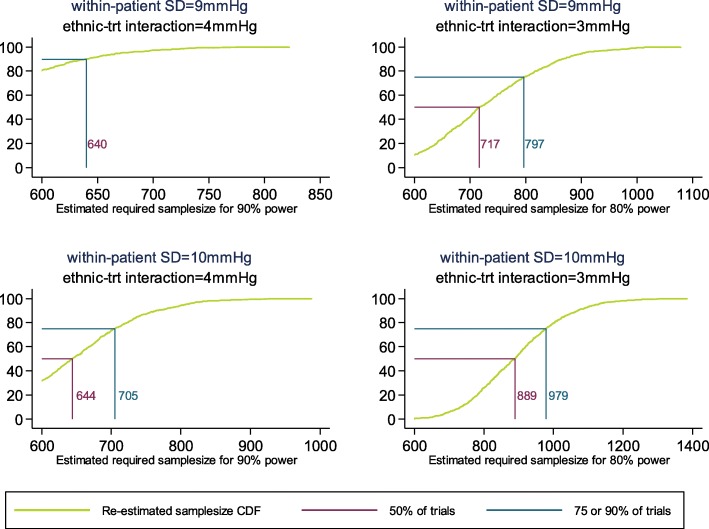
Cumulative density function for re-estimated sample size assuming 80% or 90% power to detect treatment-by-ethnic interactions using a global test of interaction at the 5% significance level for participants on the monotherapy treatment regime

In the trial protocol, the sample size calculation fixed the SD to be 8 mmHg, giving > 80% power with 600 participants. With simulations, sampling variation means that we have a range of possible estimates of within-person SD which means that in this case 90% of the time 640 participants would usually be fine to give us 90% power to detect the primary effect for a within person SD of 9 mmHg and a single treatment-by-ethnic interaction of 4 mmHg (Fig. 3). A larger within-person SD of 10 mmHg would mean that in 75% of the time 705 participants would usually be fine to give us 90% power to detect the same planned treatment effect (Fig. 3).

Assuming a smaller single treatment-by-ethnic interaction of 3 mmHg, and a 1 mmHg increase in the assumed within-person SD, 75% of the time with a sample size of 797 participants would give us 80% power to detect the primary hypothesis. A 2 mmHg increase in within-person SD from that assumed in the protocol means that 75% of the time with a sample size of 979 participants would give us 80% power detect a treatment effect of 3 mmHg (Fig. 3). As would be expected, smaller values of *δ*, and larger values of *σ*_*e*_, imply larger required sample sizes. Figure 3 shows for the scenario with *σ*_*e*_ = 9 mmHg and *δ* = 4 mmHg more than the planned 600 particpiants are required because of the nature of sample size re-estimation and the methodology used here. The conservative approach adopted here to try and control the type-I error rate means we have to push the sample size up a little to keep the type-II error rate down. That is, the use of Kenward–Roger and the Hotelling adjustments pushes the power down compared to the methodology used for the initial 600 estimate, so it is inevitable that the simulations here produce > 600 for the sample size re-estimation designs.

### Simulation of the interim analysis and re-estimation of the sample size: dual therapy

**Fig. 4:**
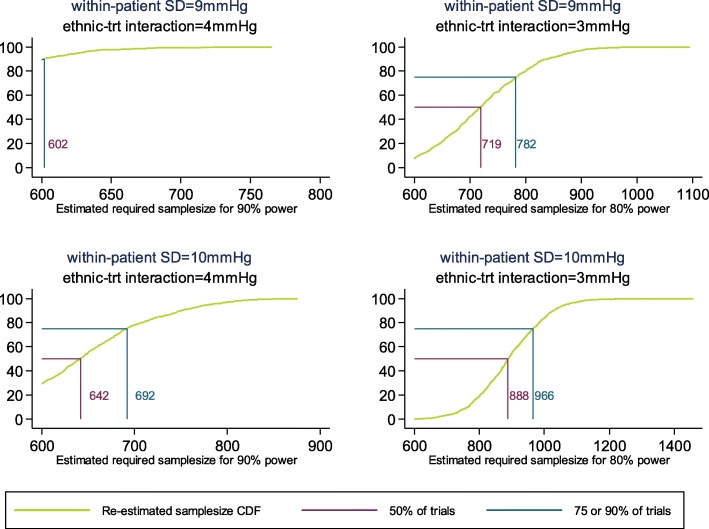
Cumulative density function for re-estimated sample size assuming 80% or 90% power to detect treatment-by-ethnic interactions using a global test of interaction at the 5% significance level for participants of the dual-therapy treatment regime

For the dual-therapy trial, sample sizes required for the different simulation scenarios are similar to those estimated for the monotherapy trial. For 90% power to detect a treatment effect of 4 mmHg, 90% of the time 602 participants would usually be fine when the within-person SD is 9 mmHg. A larger within-person SD of 10 mmHg would mean that 75% of the time 692 participants would usually be fine to give us 90% power to detect the same treatment effect (Fig. 4). In both scenarios, the dual-therapy trial requires slightly fewer participants.

Assuming a smaller single treatment-by-ethnic interaction of 3 mmHg, a 1 mmHg increase in the assumed within-person SD 75% of the time a sample size of 782 participants would give us 80% power to detect the primary hypothesis. A 2 mmHg increase in within-person SD from that assumed in the protocol means that 75% of trials with a sample size of 966 participants would give us 80% power detect a planned treatment effect of 3 mmHg; again, in both scenarios, fewer participants are required than in the monotherapy trial, as a result of having more measurements overall in the dual-therapy trial.

In summary, for the same simulated treatment-by-ethnic interaction an increase in within-person SD requires a large sample size. For the same simulated within-person SD, a smaller planned treatment effect also requires a larger sample size, which is what would be expected from sample size calculation theory. The fact that the dual-therapy trial when compared with the monotherapy trial requires slightly fewer participants when comparing like-for-like is a result of the increased number of denominator degrees of freedom in the sample size calculation for the four-period four-treatment compared with the three-period three-treatment crossover: 3n – 14 = 136 compared with 2n – 10 = 90 when *n* = 50. The increased number in the denominator degrees of freedom in the four-period four-treatment compared with the three-period three-treatment crossover is in turn due to the increased number of observations per participant in the four-by-four crossover trial.

## Discussion

A novel method for sample size re-estimation has been described for three-period three-treatment and four-period four-treatment crossover trials. Here we have dealt with a more complicated covariance matrix in a 3 × 3 and 4 × 4 randomised crossover setting that incorporates both a global test and allows for interaction terms in the linear mixed model.

The simulation study allowed us to explore the outcome for a possible increase in the within-person SD from that assumed and used for the sample size calculations in the protocol ahead of trial recruitment and the interim analysis. As would be expected, an increase in the within-person SD or a smaller primary treatment effect would require a larger sample size. The fact that the dual-therapy trial requires slightly fewer participants than the monotherapy trial when all variables are like for like is a consequence of the increased degrees of freedom in the denominator of the *F-*test which is used in the sample size calculation. The simulation of the interim sample size indicates that we can only realistically aim for 80% in these scenarios without increasing the sample size above 600 participants.

## Conclusions

The formulas presented here provide a means for re-estimating the sample size in both three-period three-treatment and four-period four-treatment crossover trials. In the context of the AIM HY-INFORM study, simulating the planned interim analysis allows us to explore the outcome for a possible increase in the within-person SD from that assumed in the protocol. Simulations show that without increasing the planned sample size of 600 participants, on each crossover trial, we can reasonably still expect to achieve 80% power with a small increased in the within person SD from that assumed.

### Supplementary information

**Additional file 1.** Matlab code.

**Additional file 2.** Stata do file.

**Additional file 3.** Three-period three-treatment sample size calculation code (Stata ado file).

**Additional file 4.** Four-period four-treatment sample size calculation code (Stata ado file).

## Data Availability

The datasets generated and analysed during the current study are available from the corresponding author on reasonable request.

## Appendix

Here, we justify our specified values for $\hat{\mathbf{Cov}}\left(\hat{\boldsymbol{\theta}}\right)$, used in the sample size re-estimation procedures. To begin, consider the 3 × 3 trial and cast our unrestricted linear mixed model in the form

$$ \boldsymbol{Y}=\boldsymbol{X}\boldsymbol{\beta } +\boldsymbol{Zb}+\boldsymbol{\epsilon}, $$

where ***β*** = (*μ*, *π*_2_, *π*_3_, *τ*_*B*_, *τ*_*C*_, *e*_2_, *e*_3_, *θ*_*B*2_, *θ*_*B*3_, *θ*_*C*2_, *θ*_*C*3_)^⊤^. Then, the maximum likelihood estimator of the fixed effects ***β*** at the end of trial is

$$ \hat{\boldsymbol{\beta}}={\left(\hat{\mu},{\hat{\pi}}_2,{\hat{\pi}}_3,{\hat{\tau}}_B,{\hat{\tau}}_C,{\hat{e}}_2,{\hat{e}}_3,{\hat{\theta}}_{B2},{\hat{\theta}}_{B3},{\hat{\theta}}_{C2},{\hat{\theta}}_{C3}\right)}^T={\left({\boldsymbol{X}}^T{\boldsymbol{\Sigma}}^{-1}\boldsymbol{X}\right)}^{-1}{\boldsymbol{X}}^T{\boldsymbol{\Sigma}}^{-1}\boldsymbol{Y}, $$

with **Σ** = ***Z*****Cov**(***u***)***Z***^*T*^ + **Cov**(***ϵ***). We then have

$$ \mathbf{Cov}\left(\hat{\boldsymbol{\beta}}\right)={\left({\boldsymbol{X}}^T{\boldsymbol{\Sigma}}^{-1}\boldsymbol{X}\right)}^{-1}. $$

See, for example, Fitzmaurice et al. [18] for further details.

In our case, $\mathbf{Cov}\left(\boldsymbol{u}\right)={\sigma}_b^2{\boldsymbol{I}}_n$ and $\mathbf{Cov}\left(\boldsymbol{\epsilon} \right)={\sigma}_e^2{\boldsymbol{I}}_{3n}.$ This implies that **Σ** is block diagonal, with *n* blocks of the 3 × 3 matrix **Σ**_3_, say, where

$$ {\boldsymbol{\Sigma}}_3=\left(\begin{array}{ccc}{\sigma}_e^2+{\sigma}_b^2& {\sigma}_b^2& {\sigma}_b^2\\ {}{\sigma}_b^2& {\sigma}_e^2+{\sigma}_b^2& {\sigma}_b^2\\ {}{\sigma}_b^2& {\sigma}_b^2& {\sigma}_e^2+{\sigma}_b^2\end{array}\right). $$

It can then be shown by verifying ${\boldsymbol{\Sigma}}_3{\boldsymbol{\Sigma}}_3^{-1}={\boldsymbol{I}}_3$ that

$$ {\boldsymbol{\Sigma}}_3^{-1}=\frac{1}{\sigma_e^2\left({\sigma}_e^2+3{\sigma}_b^2\right)}\left(\begin{array}{ccc}{\sigma}_e^2+2{\sigma}_b^2& -{\sigma}_b^2& -{\sigma}_b^2\\ {}-{\sigma}_b^2& {\sigma}_e^2+2{\sigma}_b^2& -{\sigma}_b^2\\ {}-{\sigma}_b^2& -{\sigma}_b^2& {\sigma}_e^2+2{\sigma}_b^2\end{array}\right). $$

Now

$$ {\displaystyle \begin{array}{c}\mathbf{Cov}\left(\hat{\beta}\right)={\left({X}^T{\boldsymbol{\Sigma}}^{-1}X\right)}^{-1},\\ {}={\left(\frac{n}{9}\sum \limits_{k=1}^6\sum \limits_{l=1}^3{X}_{kl}^T{\boldsymbol{\Sigma}}_3^{-1}{X}_{kl}\right)}^{-1},\end{array}} $$

where ***X***_*kl*_ is the design matrix for a single individual on treatment *k*, of ethnicity *l*.

Thus $\mathbf{Cov}\left(\hat{\boldsymbol{\beta}}\right)$ can be found by evaluating the above expression, which can be readily achieved computationally using a symbolic algebra package. Performing these calculations in Matlab, and extracting the sub-component corresponding to $\hat{\boldsymbol{\theta}}$, we identified that

$$ \mathbf{Cov}\left(\hat{\boldsymbol{\theta}}\right)=\frac{12{\upsigma}_e^2}{n}\left(\begin{array}{cccc}1& 0.5& 0.5& 0.25\\ {}0.5& 1& 0.25& 0.5\\ {}0.5& 0.25& 1& 0.5\\ {}0.25& 0.5& 0.5& 1\end{array}\right). $$

It is for this reason that we utilise

$$ \hat{\mathbf{Cov}}\left(\hat{\boldsymbol{\theta}}\right)=\frac{12{\hat{\sigma}}_e^2}{n}\left(\begin{array}{cccc}1& 0.5& 0.5& 0.25\\ {}0.5& 1& 0.25& 0.5\\ {}0.5& 0.25& 1& 0.5\\ {}0.25& 0.5& 0.5& 1\end{array}\right), $$

in our re-estimation procedure.

Equivalent calculations for the 4 × 4 trial also demonstrate that

$$ \mathbf{Cov}\left(\hat{\boldsymbol{\theta}}\right)=\frac{12{\sigma}_e^2}{n}\left(\begin{array}{cccccc}1& 0.5& 0.5& 0.5& 0.25& 0.25\\ {}0.5& 1& 0.5& 0.25& 0.5& 0.25\\ {}0.5& 0.5& 1& 0.25& 0.25& 0.5\\ {}0.5& 0.25& 0.25& 1& 0.5& 0.5\\ {}0.25& 0.5& 0.25& 0.5& 1& 0.5\\ {}0.25& 0.25& 0.5& 0.5& 0.5& 1\end{array}\right). $$

## Abbreviations

ABPM: Ambulatory blood pressure monitoring (mmHg)
AIM HY-INFORM: ComparIsoN oF Optimal HypeRtension RegiMens (Part of the Informative Markers in Hypertension (AIM HY) Programme – AIM HY-INFORM)
SBP: Systolic blood pressure (mmHg)
